# Somatic Growth Rates of Juvenile Green Sea Turtles (*Chelonia mydas*) in the Fijian Archipelago

**DOI:** 10.1002/ece3.72780

**Published:** 2026-01-14

**Authors:** Garrett E. Lemons, Calandra N. Turner Tomaszewicz, Shritika Prakash, Katy Miller, Jeffrey A. Seminoff, Susanna Piovano

**Affiliations:** ^1^ NOAA‐NMFS Southwest Fisheries Science Center La Jolla California USA; ^2^ School of Agriculture, Geography, Environment, Ocean and Natural Sciences The University of the South Pacific Suva Fiji; ^3^ Vatuvara Foundation Suva Fiji

**Keywords:** *Chelonia mydas*, Fiji, GAMM modeling, sea turtle, South Pacific

## Abstract

Establishing key life history traits (i.e., somatic growth rates) for sea turtles produces insights into population demography and informs conservation efforts. Despite a plethora of studies on sea turtles over the past decades, there remain significant knowledge gaps for the demography of many populations. From 2015 to 2022, we measured somatic growth for 215 foraging green turtles ( *Chelonia mydas* ) captured among three foraging areas in the Fijian Archipelago, tropical South Pacific. We modeled a mean size‐specific growth rate function for this foraging aggregation that was non‐monotonic decreasing with size. The mean growth rate for this foraging aggregation was 1.6 ± 0.1 cm year^−1^ curved carapace length, and we found some spatial variation in growth rates across the three foraging sites (range of means = 1.1–1.8 cm year^−1^), perhaps owing to differences in habitat quality and/or ontogeny‐based differences in feeding ecology. Overall, the range of Fijian juvenile green turtle growth rates aligns with those reported from foraging aggregations elsewhere in the Pacific and also conforms to the general pattern of non‐monotonic declining growth reported for green turtles throughout this ocean basin. Establishing foraging area‐specific growth parameters for Fijian green turtles provides current estimates to inform ecological and health assessments vital to the development of future conservation plans.

## Graphical abstract

We measured somatic growth rates of 215 juvenile green turtles across three foraging sites in the Fijian Archipelago from 2015 to 2022, finding a mean growth rate of 1.6 ± 0.1 cm year^−1^ that declined non‐monotonically with size and varied spatially between sites. These growth rates are consistent with patterns observed in other Pacific green turtle populations, reflecting influences of habitat quality and feeding ecology. Our findings establish key life history parameters for Fijian green turtles that will support future ecological assessments and conservation planning.

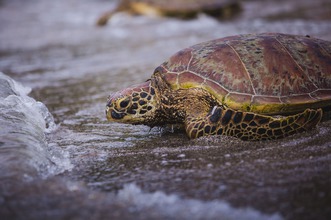

## 1 Introduction

Understanding life history and demographic parameters of endangered sea turtle populations is critical for their management and recovery. Information about somatic growth, for example, can help define life stage duration, age‐at‐maturation, and generation length for turtles assembled in local foraging areas (Carr and Goodman 1970; Bell et al. 2019; Meylan et al. 2022); data on these parameters are integral for determining stage‐based survivorship and population viability (Mazaris et al. 2006; Piacenza et al. 2017). When compared across sites for a species, somatic growth data also can reflect spatial differences in habitat quality and nutrient uptake by consumers (Bjorndal and Bolten 1988; Diez and van Dam 2002; Bell et al. 2019), which can help shape conservation priorities and actions. As a result, assessment of somatic growth should be standard practice for studies of conservation‐dependent sea turtle populations.

**FIGURE 1:**
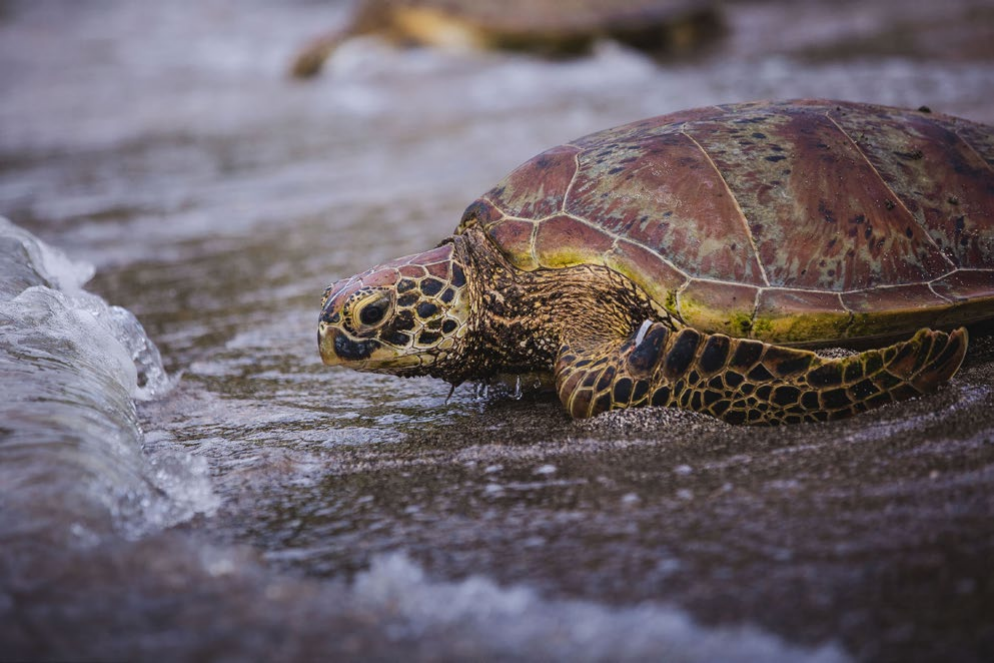
Juvenile green turtle ( *Chelonia mydas* ; locally known as *vonu*) captured, marked, measured and released at Kanacea Island during a 2022 survey. Photo made available by. Vatuvara Foundation and Zoomfiji.

The green turtle ( *Chelonia mydas* ; Figure 1) is a pantropical species that has a life history characterized by transitions among various oceanic and neritic habitats throughout its lifespan (Plotkin 2003). After hatching, green turtles emerge from nesting beaches and enter pelagic oceanic environments for approximately 3 to 8 years (Reich et al. 2007; Turner Tomaszewicz et al. 2022), eventually recruiting to nearshore developmental habitats as larger immature turtles (Shimada et al. 2014; Turner Tomaszewicz et al. 2018, 2022). Upon entering neritic habitats, green turtles may encounter a variety of habitat types with differing biotic and abiotic features, which can result in disparate resource availability across localities within a region. Whereas green turtles are thought to choose sites with greater food availability (Fujisaki et al. 2016; Griffin et al. 2020), other factors such as predation threats (Heithaus et al. 2007), shelter availability, and competition also influence habitat selection (Schofield et al. 2022; Lamont et al. 2023). The result is that green turtles may occupy habitats with fewer food resources, and remain in such areas because of greater survival potential (e.g., Rezaie‐Atagholipour et al. 2021). Because sea turtle somatic growth is strongly influenced by rates of nutrient uptake (Bjorndal et al. 2000; Omeyer et al. 2018), turtles in habitats with differing food quality and availability may grow at different rates and achieve differential levels of fitness (Lamont and Johnson 2021). Moreover, local density‐dependent factors present in some foraging areas may result in slower growth of juveniles assembled there (Mortimer et al. 2025). Indeed, as has been shown for both sea turtles and freshwater turtles, subtle individual variation in somatic growth in juvenile age classes can lead to major differences in individual survival and lifetime reproductive output (Bjorndal et al. 2013; Armstrong et al. 2018).

One area where green turtles live in diverse marine habitat types within close proximity is the Fiji Islands (Piovano et al. 2019). Consisting of more than 300 islands, Fiji harbors high marine biodiversity spread over an enormous area of reef habitat that represents nearly 3.5% of the total area of coral reefs in the world (Feussner et al. 2012) and also has extensive seagrass communities throughout the country (Singh 2019). Green turtles inhabiting Fijian waters originate from nesting beaches throughout the South Pacific (Guinea 1993; Craig et al. 2004; Piovano et al. 2019), with mixed stock analysis indicating a majority of genetic contribution coming from the American Samoa Management Unit (Piovano et al. 2019). Once a prized food and cultural resource locally but now under legal protection (Guinea 1993; Republic of Fiji 2014; Kitolelei et al. 2022), foraging turtles have been documented at several different localities (Batibasaga et al. 2006; Sykes et al. 2018; Piovano et al. 2020). However, despite the importance of Fiji as a key foraging destination for green turtles from throughout the South Pacific region, little is known about the population biology and demography of turtles foraging in these islands.

Here we evaluate somatic growth rates of juvenile green turtles foraging at three islands in the Fijian Archipelago: Kanacea Island, Makogai Island, and Yadua Island. We collected data via mark‐recapture and evaluated the effect of body size, recapture interval, year, and location on growth rates using a generalized additive mixed‐effect model. We discuss the patterns of somatic growth for green turtles in Fiji and explore juvenile stage duration at these sites and within size classes. We also compare our findings with other green turtle populations across the tropical and sub‐tropical Pacific. This is the first study to establish key baseline demographic parameters for green turtles foraging in the Fijian islands and provides critical information to advise future green turtle assessments and management plans in the region.

## 2 Methods

### 2.1 Study Sites

**FIGURE 2:**
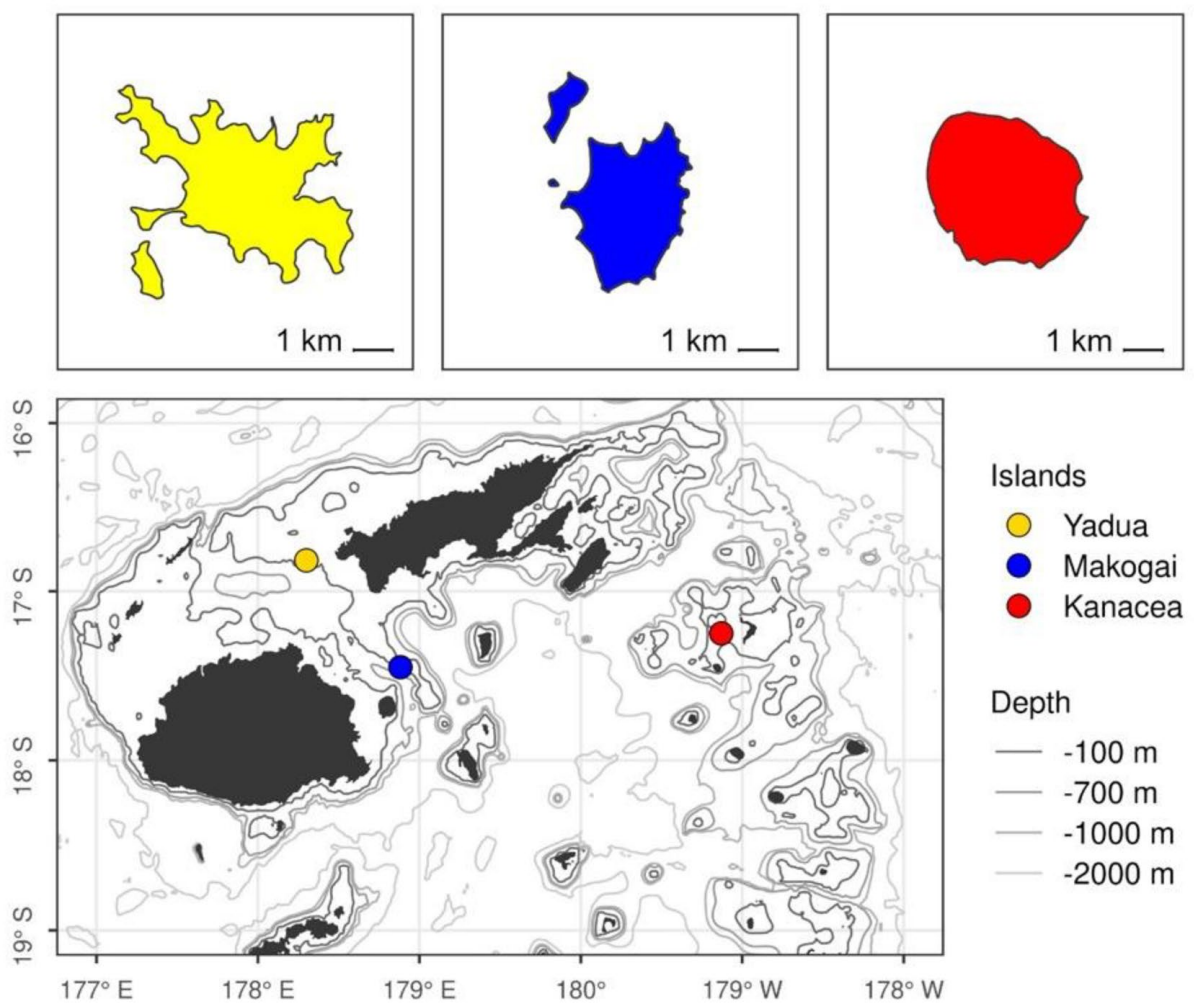
Map of Fijian Island archipelago depicting location of the three study sites for the present study: Yadua Island (yellow), Makogai Island (blue), and Kanacea Island (red).

In‐water, mark‐recapture surveys were carried out at coastal foraging grounds of Yadua Island (16.8167° S, 178.2996° E), Makogai Island (17.4473° S, 178.9653° E), and Kanacea Island (17.2589° S, 179.1468° W) (Figure 2). Yadua and Makogai Islands are located at the respective northwest and southeast ends of the Vatu‐i‐Ra Channel between the larger main Fijian islands of Viti Levu and Vanua Levu (Figure 2). Kanacea lies northeast of Makogai, ~200 km southeast off the coast of Vanua Levu and is considered part of the Northern Lau Island group. All three study sites are neritic habitats varying in seagrass meadows and algal beds that are adjacent to further offshore barrier and/or fringing coral reef habitats and reef channels (McKenzie and Yoshida 2020; Papale et al. 2020; Piovano et al. 2020), These reef channels ultimately influence the rate of inner water turnover (Breckwoldt et al. 2022), thus influencing coastal habitats that host year‐round foraging aggregations of juvenile green turtles (Piovano et al. 2019, 2020). Among the three study sites, Yadua Island has been reported to contain the greatest benthic coverage of seagrass (up to 46% coverage), with the majority being *Halodule pinifolia* (Piovano et al. 2020), whereas Makogai Island has up to ~28% benthic coverage, primarily of *Halodule uninervis* (Papale et al. 2020). Benthic survey information for Kanacea Island is unavailable.

### 2.2 Green Turtle Capture and Data Collection

At least one four‐day capture‐mark‐recapture survey was conducted annually at each island site within reef and shallow lagoon areas (max –30 m; GEBCO 2023) during the study durations. Specific depths and years of capture for each island varied, with Kanacea Island captures occurring from 2019 to 2022 at depths of 2–12 m, Makogai Island captures occurring from 2015 to 2016 and 2018 to 2020 at a depth of ~4 m, and Yadua Island captures occurring from 2015 to 2022 at a depth of ~4 m. During each survey, turtles were hand‐captured at high tide by swimmers in the water, as detailed in Ehrhart and Ogren (1999). Minimum curved carapace length (CCL) was measured from the nuchal notch to the posterior‐most edge of the rear marginal scutes using a flexible measuring tape (Bolten 1999). Captured turtles were checked for the presence of external injuries and fibropapilloma tumors. Each turtle had a uniquely coded Inconel flipper tag (National Band and Tag Co., Style 681) applied to the trailing edge of either one (up to February 2020) or both (from March 2020) front flippers (Eckert and Beggs 2006) upon initial capture. Damaged or loosely fitting tags encountered during recapture events were removed and replaced.

### 2.3 Somatic Growth Rates and Data Analysis

For individual turtles, simple linear growth rates were calculated by interpolation of consecutive CCL measurements from individuals that had a total recapture interval (R_i_) of at least 270 days (Chaloupka and Musick 1997). Our study employed a mixed longitudinal sampling design, and we note that age and cohort effects are unknown and thus confounded in the sampling design (Chaloupka and Musick 1997). Individual turtles with more than two growth increments (individuals re‐captured more than once) were treated as random effects in the model, and individual variation was modeled with a random intercepts term. Therefore, we modeled growth rates with a generalized additive mixed‐effect model (GAMM)—we modeled overall growth and for each foraging location (Kanacea, Makogai, and Yadua Islands).

The generalized additive mixed‐effect model is defined by the following equation:

$$g\left(Y\right)=\alpha +s_{1}\left(x_{1}\right)+\ldots +\mathrm{sp}\left(\mathrm{xp}\right)$$

where *Y* is the response variable or growth rate, *g* denotes the link function used, *α* denotes the dependent parametric terms and the terms *s*_1_(*x*_1_) + … + *sp*(*xp*) denote smooth non‐parametric functions. GAMM modeling used a Gaussian identity link function with cubic regression smoothing splines and incorporated a quasi‐likelihood error estimate (S. N. Wood 2004). Our response variable was growth rate. Non‐parametric independent variables for model selection included mean CCL and recapture interval (*R*_*i*_ hereafter). Mean CCL was the average of the initial CCL measurement and the CCL measurement at subsequent capture. *R*_*i*_ is defined as the number of days between initial and final captures, or the interval between subsequent captures for individuals sampled repeatedly. Model parametric terms included foraging location (Kanacea, Makogai, or Yadua islands) and year of growth defined as the median calendar year between measurements. We note that year as a source of growth variability is confounded with cohort effects because of the unknown ages of turtles inherent to this study design. We acknowledge the potential imprecision of using per‐year growth rate due to intra‐annual (i.e., seasonal) growth disparities when applied to turtles with an *R*_*i*_ > 1 year. For the turtles with an *R*_*i*_ covering multiple years, year of growth was defined as the year as the midpoint between subsequent captures (*R*_*i*_ values; Table 1). To determine significance of parametric terms, ANOVA was used to examine green turtle somatic growth rates by location and year and a Tukey post hoc analysis was applied to identify where the parametric parameters varied. All statistical analyses were conducted using R (R Core team 2024) and the package mgcv (S. N. Wood 2004, 2023).

**TABLE 1:** Summary of green turtle growth rates and model covariates at three study areas in Fiji. CCL is defined as curved carapace length and R_i_ is defined as time at large.

Island	N_i_	N_c_	N_e_	Mean growth rate ± SE (cm year^−1^)	Range (cm year^−1^)	Mean CCL ± SE (cm)	Range (cm)	R_i_ ± SE (days)	Range (days)
Yadua	104	222	120	1.8 ± 0.2	0.0–7.9	53.1 ± 0.6	43.5–77.8	686 ± 35.7	187–1643
Makogai	22	51	31	1.3 ± 0.3	0.0–5.7	57.6 ± 1.2	47.3–77.7	510 ± 76.8	39–1196
Kanacea	86	178	101	1.1 ± 0.3	0.0–5.2	62.1 ± 1.8	45.8–104.0	377 ± 28.0	116–1236
TOTAL	212	468	252	1.6 ± 0.1		56.6 ± 0.7		657 ± 27.3	

*Note:* Values are expressed as means followed by ± standard error (SE) with the ranges reported just below. N_i_ represents total number of individual animals sampled, N_c_ represents the total number of body size measurements, and N_e_ represents the number of growth estimates including recaptures.

### 2.4 Stage Duration

We estimated stage duration for four CCL classes (< 50.0 cm; 50.0–59.9 cm; 60.0–69.9 cm; ≥ 70 cm) and at each island location (Figure 4, Table 2) by dividing the CCL range of the selected size class (or island) by the mean growth rate for the respective size class (or island). For size classes < 50 and ≥ 70, ranges used in calculations were 38.0–49.9 and 70.0–104.0 cm CCL, respectively. Our size class grouping structure follows previously published growth studies on green sea turtles (Limpus and Walter 1980; Seminoff et al. 2002; Balazs and Chaloupka 2004; Sampson et al. 2015; Zárate et al. 2015), which enhances the comparability across studies.

**TABLE 2:** Summary of parameter estimates for selected GAMM growth model.

GAMM model	Parametric coefficients					Non‐parametric coefficients			
	Variable	Estimate	Std. Error	*t*	*p*	Variable	edf	*F*	*p*
Overall	(intercept)	−168.5	131.8	−1.3	0.2	s(mean_CCL_)	1.91	1.43	0.25
	Island	1.04	0.21	4.81	**0.0005**	s(R_i_)	1.43	1.01	0.50
	Year	0.08	0.06	1.3	0.2				
Kanacea Island	(intercept)	21.38	248.36	0.09	0.93	s(mean_CCL_)	1	1.57	0.21
	Year	−0.01	0.12	−0.08	0.93	s(R_i_)	3.23	2.7	**0.03**
Makogai Island	(intercept)	−161.6	749.88	−0.21	0.83	s(mean_CCL_)	1	3.94	0.05
	Year	0.08	0.37	0.21	0.83	s(R_i_)	3.05	1.43	0.21
Yadua Island	(intercept)	−201.08	152.6	−1.13	0.19	s(mean_CCL_)	3.55	5.1	**0.002**
	Year	0.1	0.07	1.33	0.18	s(R_i_)	1	0.01	0.94

*Note:* Results are presented for all four models—(i) overall population (ii) Kanacea Island (iii) Makogai Island and (iv) Yadua Island. Explanatory variables are denoted as either parametric (Island and Year) or non‐parametric (mean size and recapture interval; s(mean_CCL_, *R*_*i*_ respectively)). For parametric coefficients estimate value is the intercept value when all predictors are set to zero, the Std. Error provides a measure of the uncertainty in the estimated coefficient, the *t*‐value represents how many standard errors the estimate is from zero. For the non‐parametric coefficients edf represents the effective degrees of freedom, *F*‐statistic represents how well the smoothed terms fit the data. *p* values determine parameter significance (bold values are significant).

## 3 Results

### 3.1 Turtle Capture and Sizes

From 2015 to 2022, 215 individual green turtles were captured at least twice, resulting in 466 body size measurements producing 251 growth rate estimates (31 of the 215 individuals were captured three or more times; Table 1). The overall mean ± SD *R*_*i*_ was 657 ± 27.3 days with a minimum *R*_*i*_ of 270 days and a maximum of 1750 days (Table 1). For Yadua Island the mean *R*_*i*_ was 686 ± 35.7 days with a minimum of 270 days and a maximum of 1750 days (Table 1). For Makogai Island the mean *R*_*i*_ was 510 ± 76.8 days with a minimum of 270 days and a maximum of 1205 days. At Kanacea Island, the mean *R*_*i*_ was 377 ± 28.0 days with a minimum of 116 and a maximum of 1236 days.

**FIGURE 3:**
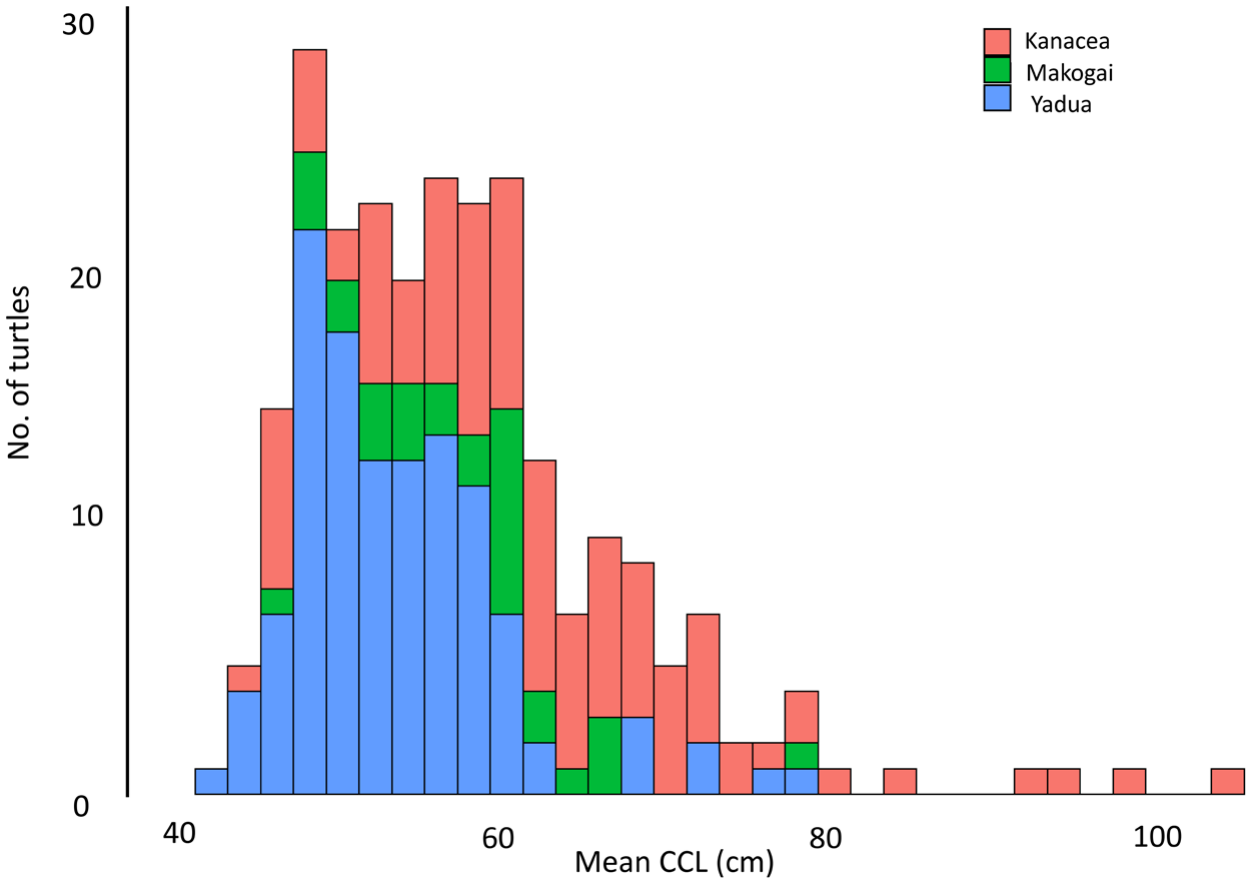
Mean curved carapace lengths (CCL; cm) for foraging green turtles at the study sites in Fiji: Kanacea Island (blue; *n* = 88), Makogai Island (green; *n* = 23), and Yadua Island (blue; *n* = 104). Turtles grew the fastest growth at Yadua Island.

Green turtle size distributions were established for the entire study group and for each foraging location. From 466 total body size measurements, overall CCL ranged from 41.1 to 104.0 cm with a mean size of 57.3 ± 0.7 cm (Table 1, Figure 3). Turtles at Yadua Island had a mean CCL of 53.1 ± 0.6 cm (*n* = 120), turtles at Makogai Island had a mean CCL of 57.6 ± 1.2 cm (*n* = 32), and turtles at Kanacea had a mean CCL of 62.1 ± 1.8 cm (*n* = 100) (Table 1, Figure 3). ANOVA indicated significant differences in mean CCL among locations, and post hoc analyses indicated turtles foraging at Yadua Island (mean CCL = 53.1 ± 0.6 cm, ~4 cm below the overall mean) were significantly smaller than those foraging at Kanacea Island (mean CCL = 62.1 ± 1.8 cm, ~5 cm above the overall mean) (Table 1; Figure 3).

### 3.2 Somatic Growth Estimates

**FIGURE 4:**
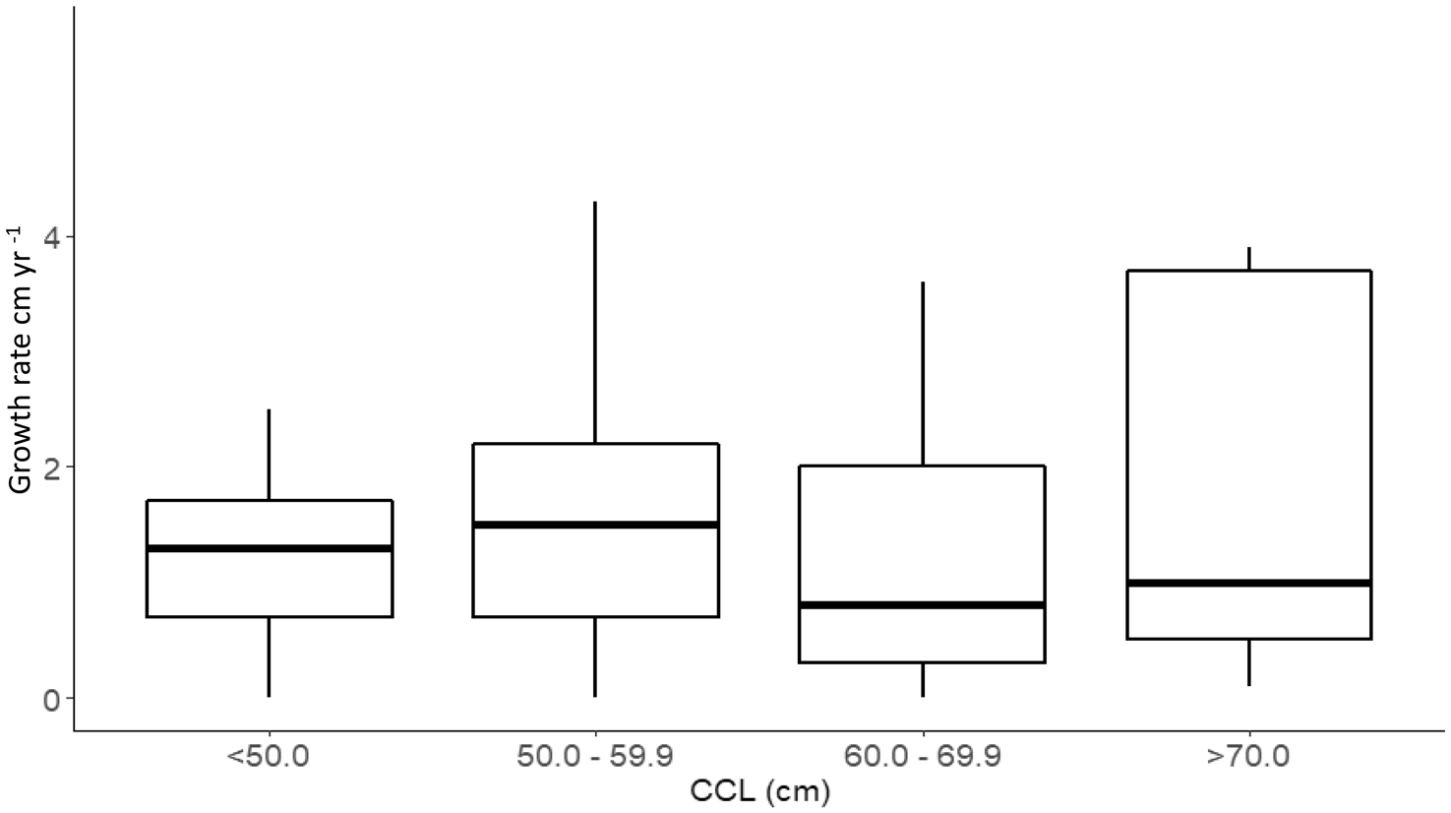
Fijian green turtle growth rates by 10‐cm curved carapace length (CCL) size class. ANOVA among size classes revealed no significant effect of size class on growth rate. Size class intervals were chosen to allow for comparisons with previous studies (Table 3). Fastest growth was in the 50–59.9 cm CCL group.

**FIGURE 5:**
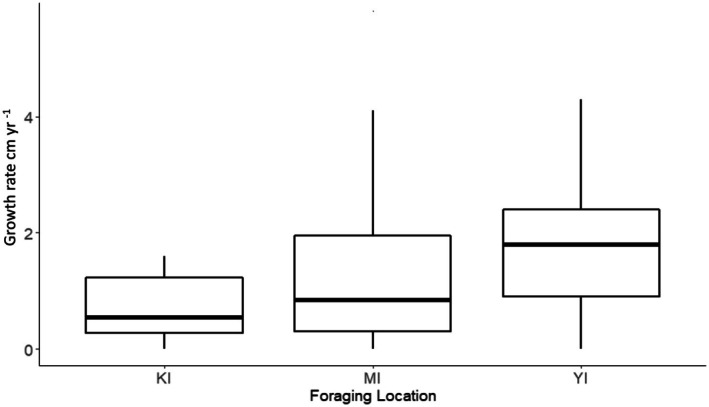
Fijian green turtle growth rates in curved carapace length (CCL) at each capture location (KI = Kanacea Island, MI = Makogai Island, YI = Yadua Island). Model results indicate a statistically significant difference in growth rate among the islands and post hoc analysis indicated significant disparity between Yadua Island and Kanacea Island, yet the mean differences are small, less than 0.5 cm (Table 1).

The mean growth rate for all foraging locations and all turtles was 1.6 ± 0.1 cm year^−1^ (*n* = 251, range = 0.0–5.7 cm year^−1^; Table 1). Site‐specific somatic growth varied across the three study sites (ANOVA, df(2), *F*(15.4)) with mean growth rates at Kanacea, Makogai, and Yadua Islands of 1.1 ± 0.3 cm year^−1^, 1.3 ± 0.3 cm year^−1^, and 1.8 ± 0.2 cm year^−1^, respectively (Table 1; Figure 5). A post hoc analysis revealed that the mean growth rate at Yadua Island (1.8 ± 0.2 cm year^−1^) was significantly faster than that for Kanacea Island (1.1 ± 0.3 cm year^−1^; Table 1; Figure 5), although we acknowledge that the actual differences from the overall mean—0.2 cm year^−1^ and 0.5 cm year^−1^, respectively—are within the margin of error when measuring CCL. Within sites, there were apparent differences in growth rates among the four size classes of turtles (Figure 4), although (ANOVA df(3), *F*(1.3)) analyses indicated these differences were not significant.

### 3.3 Growth Modeling

**FIGURE 6:**
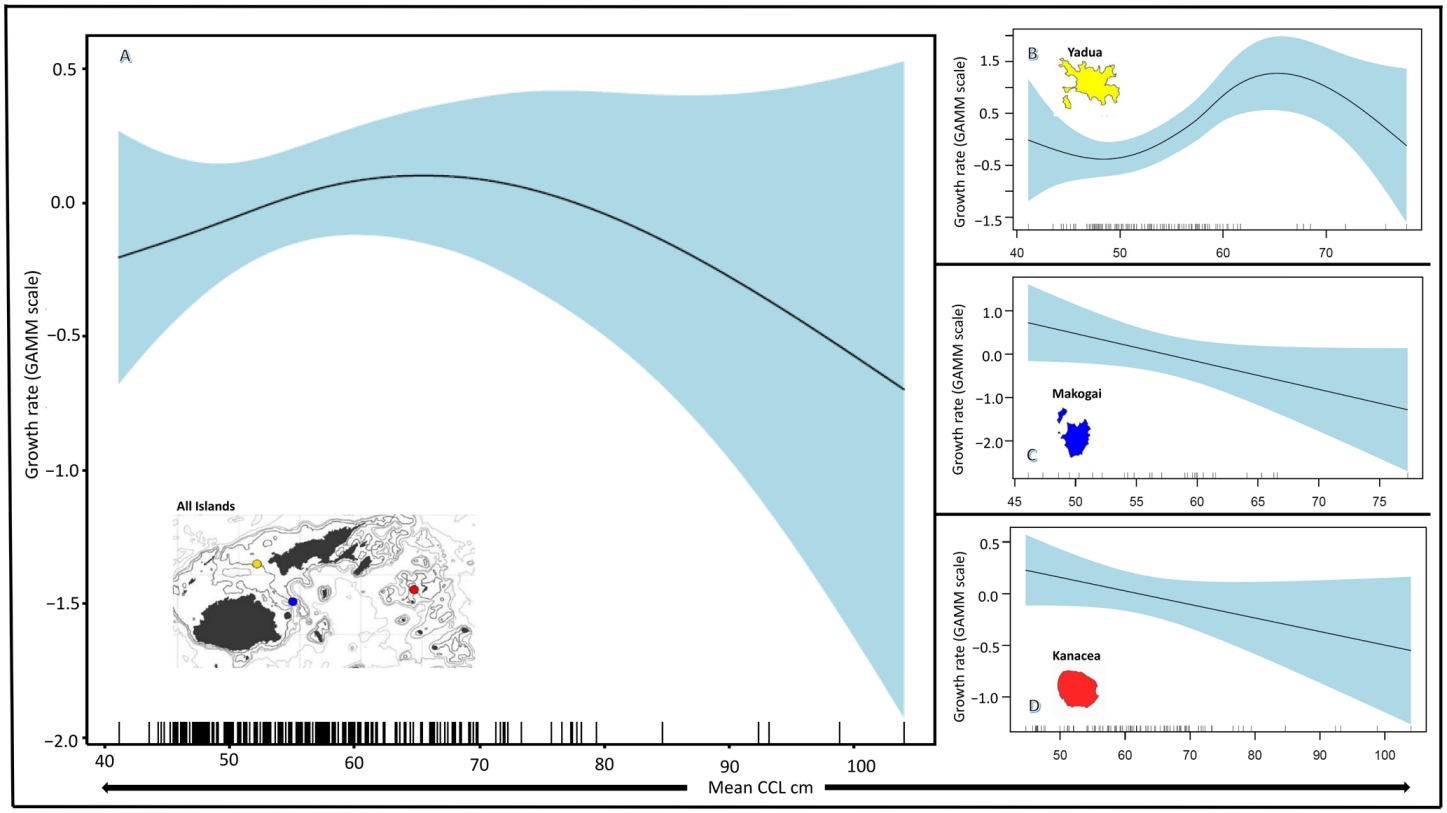
GAMM modeled green turtle growth for the overall population for the significant coefficients ‐ Island. Panel A is overall green turtle growth rate (y‐axis) modeled as a function of mean size (x‐axis) for all foraging locations in this study. Overall growth was non‐monotonic. Panels B, C, and D represent growth for Yadua Island, Makogai Island and Kanacea Island, respectively, modeled as a function of turtle size (x‐axis).

**FIGURE 7:**
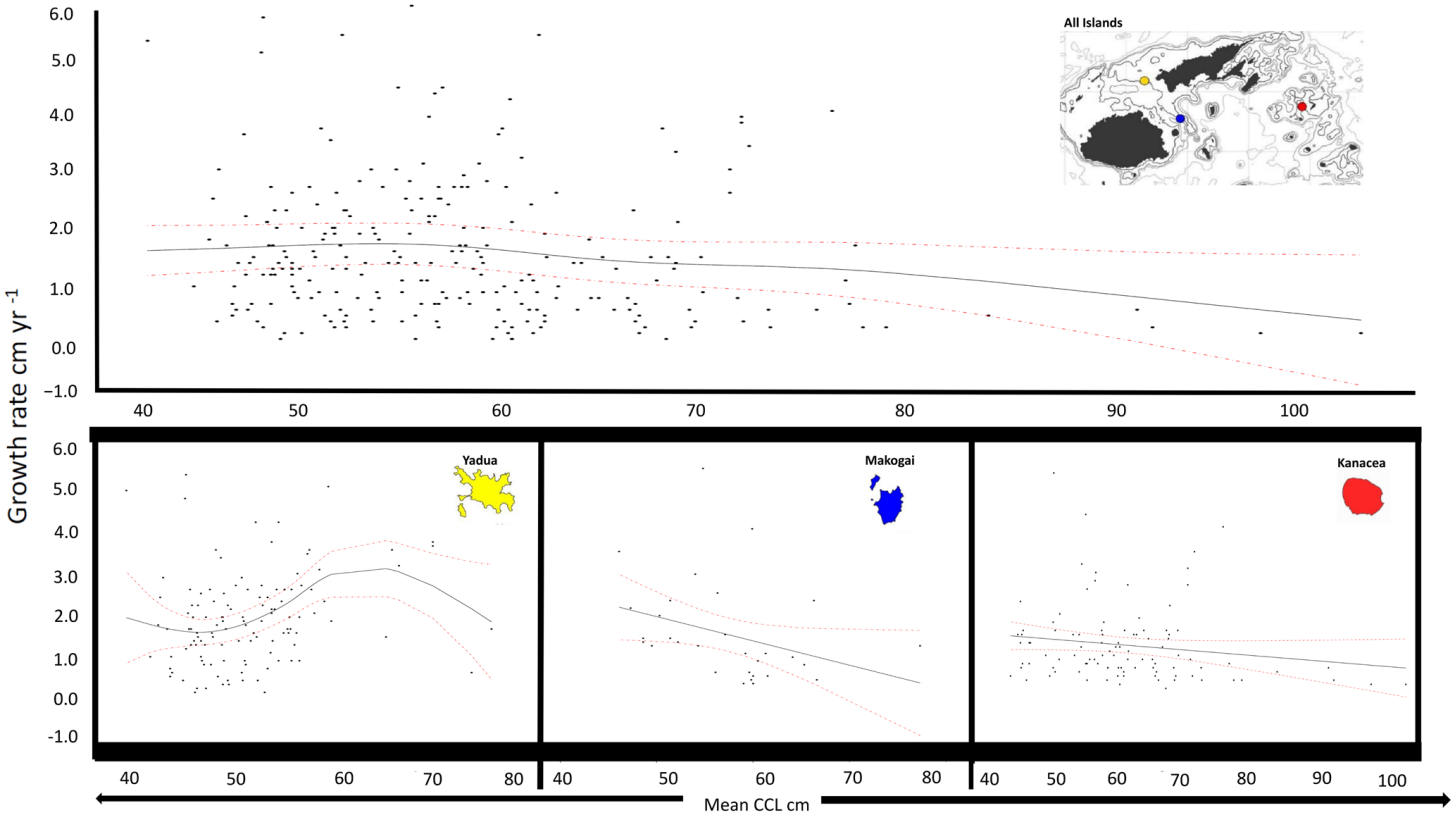
Predicted growth at size for green turtles in the Fijian Archipelago. The GAMM growth function was fitted over plotted empirical individual green turtle mean sizes vs. growth rates for all turtles (top panel) and for each island (tri panel below). Solid black line indicates predicted growth at size and red dashed lines indicated 95% C.I. for the prediction of the fitted growth function.

None of the covariates explained the variance in growth rates except for location (Table 2; Figure 6). The GAMM fit indicated a linear decrease in growth rate with an increasing *R*_*i*_, however, *R*_*i*_ was not significant in explaining growth rate variance. Likewise, year of growth did not significantly explain variance in overall green turtle growth. Subsequently, our overall model indicated the only significant coefficient for growth rate was foraging location (Table 2, Figure 6). We note that when modeled alone (growth ~ s(mean_ccl_)), turtle size significantly explained turtle growth rate, but when the model was expanded to include location (growth ~ s (mean CCL) + location), turtle size lost significance; but this model explained variation in growth more than size or location alone. Post hoc analysis revealed that the significant effect of location was a result of turtles at Yadua Island having significantly faster growth rates than Kanacea Island (Table 2; Figure 5). Overall, our model indicated a non‐monotonic pattern of growth with growth rates decreasing with turtle size influenced by spatial variability in foraging locations (Figure 6). We offer visualization of this model by predicting growth rate across the range of observed CCLs with a 95% prediction interval (Figure 7).

### 3.4 Kanacea Growth

For turtles foraging at Kanacea island (with CCLs ranging between 45.8 and 104.0 cm), none of the covariates explained the variance in growth rates well except for *R*_*i*_ (Table 2; Figure 4). Turtle size and year had no significant effect on growth for turtles at Kanacea. Although not significant, growth for turtles at Kanacea appears to be linear decreasing with increasing size (Table 2, Figure 6). *R*_*i*_ was the only significant coefficient in explaining growth variance for Kanacea turtles. Growth as a function of *R*_*i*_ for Kanacea indicated growth as monotonic decreasing as the *R*_*i*_ increases (Figure 6).

### 3.5 Makogai Growth

For turtles foraging at Makogai Island (with CCLs ranging between 47.3 and 77.7 cm), none of the covariates were significant (Table 2). For Makogai, turtle growth as a function of size is linear and decreasing. Modeling growth as a function of the *R*_*i*_ for Makogai indicated non‐significance and a linear decrease in growth rate with an increasing *R*_*i*_. Likewise, the year of growth did not significantly explain variance in green turtle growth at Makogai (Table 2). We note that while none of the model coefficients for Makogai were significant, growth as a function of size was on the border of significance (*p* = 0.05, Table 2).

### 3.6 Yadua Growth

For turtles foraging at Yadua Island (with CCLs ranging between 43.5 and 77.8 cm), size was the only covariate that explained the overall variance in growth rates, and this was the site with the smallest measured mean CCL (Table 2; Figure 3). Turtle growth as a function of size was monotonic decreasing (Figure 6). The GAMM model for the *R*_*i*_ coefficient indicated a linear decrease in growth rate with an increasing *R*_*i*_. However, *R*_*i*_ was not significant in explaining growth variance. Likewise, year of growth did not significantly explain variance in green turtle growth at Yadua Island (Table 2).

## 4 Discussion

The information on somatic growth reported here represents the first such information for green turtles in the Fijian Archipelago and, to our knowledge, also the first in the Central South Pacific, a region for which relatively little biological information is available for the species (Seminoff et al. 2015). Our analysis of green turtle growth rates revealed three main findings: (1) an overall pattern of non‐monotonic growth with maximum growth rates in intermediate‐sized (50.0–59.9 cm CCL) turtles, followed by decreasing growth with increased size, (2) spatial variation in green turtle growth dynamics among the three Fijian study sites, and (3) similar growth patterns to green turtles across the Pacific but disparate to those in the Atlantic, which are largely monotonic declining growth rates with increasing size (e.g., Bjorndal and Bolten 1988).

### 4.1 Body Size and Life Stage

Based on body sizes, it is apparent that the foraging groups at all three foraging sites are largely comprised of small to large immature turtles, although one site (Kanacea Island) included a small number of putative adult turtles (> 85 cm CCL, *n* = 4). The groups of turtles at the three study sites included small, post pelagic juveniles (minimum CCLs range from 43.5 to 47.3 cm), which are consistent with sizes of newly recruited turtles for the region and generally consistent with recruitment sizes for green turtles throughout the South Pacific (Piovano et al. 2019). The mean nesting size (MNS) of green turtles at Rose Atoll, American Samoa—the primary source rookery for green turtles in Fiji (Piovano et al. 2019)—is 101.6 cm CCL (range 85.0–114.7 cm; Murakawa et al. 2024). This suggests that the largest turtles at both Yadua Island and Makogai Island (CCLs = 77.8 cm and 77.7 cm, respectively) were large immature turtles. The Kanacea Island study group included four relatively large turtles (CCLs of 92.4 cm, 93.2 cm, 98.8 cm, and 104.0 cm; Figure 3) that were within the size range of females nesting at Rose Atoll, suggesting that all four individuals could be mature adult turtles; however, all other turtles at Kanacea Island were ≤ 84.7 cm indicative of an aggregation of small‐to‐large immature turtles (although these turtles tended to be larger immatures than found at the other sites).

The reasons for adult and generally larger immature turtles being encountered only at Kanacea Island are unclear, especially considering that all three study sites are neritic habitats containing seagrass meadows and algal beds that are adjacent to further offshore barrier and/or fringing coral reef habitats (McKenzie and Yoshida 2020; Papale et al. 2020). However, it is possible that the location of Kanacea—most distant from the main islands of Viti Levu and Vanua Levu—provides resources and/or habitat structure that is preferred by these larger size classes (Piovano et al. 2020; Figures 2 and 3). Further analysis of the oceanography, habitat structure, migratory routes, and food web dynamics across these sites is warranted to gain a better understanding of the potential drivers for green turtle presence in these areas.

### 4.2 Size‐Related Patterns of Somatic Growth

**FIGURE 8:**
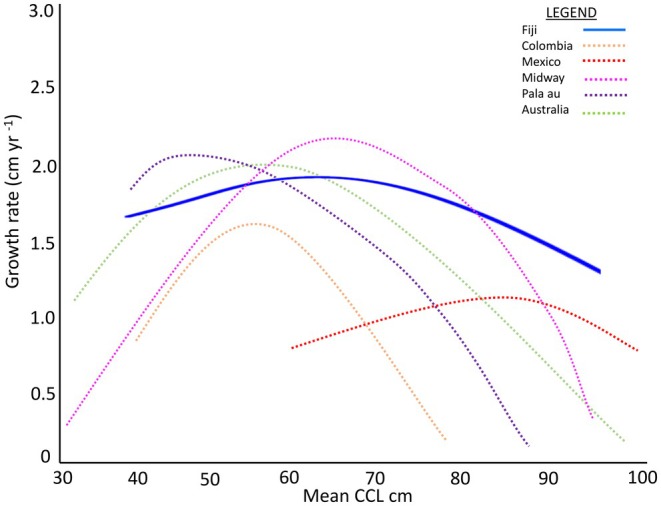
Somatic growth curves reported for green turtles across the Pacific. Key for curve line color: Fiji Archipelago (blue; this study; non‐monotonic), Pacific Colombia (orange; Sampson et al. 2015), Baja, Mexico (red; Seminoff et al. 2002), Midway Islands (pink; Balazs and Chaloupka 2004), Pala'au (purple; Balazs and Chaloupka 2004); Great Barrier Reef, Australia (green; Limpus and Chaloupka 1997).

In Fiji, we found that green turtle somatic growth as a function of body size was non‐monotonic declining, with maximal growth (i.e., a growth spurt) in an intermediate size class (50.0–59.9 cm CCL), followed by decreasing growth as body size increases (Figures 6, 7, 8). Non‐monotonic declining growth curves appear to be a common trait of green turtles in foraging areas throughout the Pacific (Figure 8). For example, green turtles in Hawaii (Balazs and Chaloupka 2004), Pacific Colombia (Sampson et al. 2015), and the Galapagos Islands (Zárate et al. 2015) also had maximum growth rates in the 50–59.9 cm CCL size class (Figure 4). However, growth spurts also occur in larger turtles: northern and southern Great Barrier Reef green turtles exhibited maximum growth rates in the 60–70 cm CCL size range (Chaloupka et al. 2004). Hawaiian foraging female green turtles exhibit peak growth in the 70.0–79.9 cm SCL range (Murakawa and Snover 2018), and the fastest growing turtles in the Gulf of California, Mexico, were in the 80–90 cm (SCL) size range (Seminoff et al. 2002). Why green turtles exhibit growth spurts remains unclear, but may relate to inherent genetic traits (Avery 1994), compensatory growth (Roark et al. 2009), and/or differential foraging strategies and nutrient intake across size classes (Quiñones et al. 2022). While exact mechanisms for variable growth remain elusive, observed variable growth in Pacific green turtles may confer several selective advantages. For example, the capacity for slower growth can reduce metabolic requirements and thus predation risk by allowing for prolonged residence in safer habitats, while the ability to grow faster enables early maturation and reproduction when resources are in abundance. Such plasticity in growth allows green turtles to optimize foraging and energy allocation across heterogenous environments and climatic regimes. This phenotypic flexibility enhances fitness under variable oceanographic conditions and likely facilitates persistence in the spatially and temporally dynamic Pacific (Balazs and Chaloupka 2004; Murakawa and Snover 2018).

In contrast to Pacific populations, size‐specific growth rates for Western Atlantic and Caribbean waters are commonly monotonic with declining growth rate as carapace length increases. In Bahamian green turtles, Bjorndal et al. (2000) reported maximum growth rates in green turtles between 30 and 40 cm SCL and minimum growth rates at around 80 cm SCL. Additional Atlantic sites where green turtles exhibit monotonic declining growth rates are summarized in Seminoff et al. (2002). Moreover, the non‐monotonic/monotonic growth function dichotomy is also present for hawksbill turtles ( *Eretmochelys imbricata* ) from the Caribbean, Indian Ocean, and southern Great Barrier Reef (Bjorndal and Bolten 1988; Chaloupka and Limpus 1997; Mortimer et al. 2025). The factors that contribute to this trend are poorly understood and caution should be exercised in formulating theories about sea turtle growth based on the few studies that have occurred to date. Harvest histories or population demography may affect size‐specific growth patterns (Bjorndal et al. 2000) as could the oceanography, recruitment and dispersal patterns at each foraging area. These variable data underscore the need for additional comparative studies of intrinsic and environmental effects on sea turtles to assess their relative influence on growth.

### 4.3 Variation in Somatic Growth Rates in Fiji

Evaluating a single mean growth rate for individuals of a wide size range assembled at a study area likely yields an overly simplistic view of local growth dynamics. However, this approach can allow for meaningful comparisons across populations at regional and ocean basin scales. Here, we found slight variation in mean growth rate across the three study sites (1.1–1.8 cm year^−1^, Table 1) that coincides with differences in mean body size of turtles in these areas. For example, the green turtle aggregation at Kanacea Island has the largest mean size (CCL = 62.1 ± 1.8 cm), including four adult‐size turtles, and has the slowest mean growth rate, whereas Yadua Island green turtles have the smallest mean size (CCL = 53.1 ± 0.6 cm) and the fastest mean growth rate. Green turtles at Makogai Island have intermediate mean CCL (57.6 ± 1.2 cm) and mean growth rate (1.3 ± 0.3 cm year^−1^) (Table 1, Figure 5), and notably, these two islands (Makogai and Yadua Island) lack any putative adult green turtles based on the measured CCLs. This inverse relationship between mean body size vs. mean somatic growth rate may relate to size‐based (i.e., intrinsic) differences in feeding ecology and nutrient uptake, or perhaps variation in extrinsic factors such as habitat quality and/or resource availability across the Archipelago. Piovano et al. (2020) found that green turtles at Yadua and Makogai Islands consumed a diverse suite of prey, suggesting there is potential for size‐ and location‐based differences in prey intake. Moreover, there have been efforts to understand marine habitat spatiotemporal variability throughout Fiji (Lawson et al. 2021), yet more information is needed about the relationship between somatic growth, habitat quality, and diet intake to better understand the drivers for the observed spatial patterns for somatic growth.

The variability in foraging site‐specific mean growth rates found in Fiji (1.1–1.8 cm year^−1^, Table 1) suggests that green turtles here may experience diverse physical and/or biological conditions at their foraging grounds. Understanding how these growth rates compare to those for green turtles at other Pacific foraging grounds (Table 3) can help depict these findings in a greater context. For example, Fijian green turtle growth rates were similar to those reported for green turtles in foraging grounds in Baja California, Mexico (mean 1.3 ± 0.8 cm year^−1^; Seminoff et al. 2002), the NW Hawaiian Islands (mean 1.1 ± 0.4 cm year^−1^; Balazs and Chaloupka 2004; Table 3) and the northern Great Barrier Reef, Australia (growth rate 1.3–2.1 cm year^−1^; Limpus and Chaloupka 1997, Chaloupka et al. 2004; Table 3; Figure 8). However, green turtles in this study grew substantially slower than those in the main Hawaiian Islands (median 3.4 cm year^−1^; Balazs and Chaloupka 2004; Table 3; Figure 8) yet noticeably faster than green turtles in Pacific Colombia (0.74 ± 0.93 cm year^−1^; Sampson et al. 2015) and especially the Galapagos Islands, where green turtles apparently grow the slowest for anywhere in the Pacific (maximum growth = 0.4 ± 0.5 cm year^−1^ in the 50–60 cm SCL size class, Zárate et al. 2015; Table 3). In Galapagos, the slow growth is attributed to high turtle densities at the foraging grounds (Green 1993; Zárate et al. 2015) and the consequential low food availability. However, why green turtles grow so fast in Hawaii is less clear. These course‐scale comparisons of somatic growth among Pacific sites at least suggest that the habitat conditions in Fiji are somewhat favorable for local green turtles, even at the site (Kanacea Island) with the slowest growing turtles.

**TABLE 3:** Green turtle mean growth rates (CCL cm/year) by carapace length class at study sites throughout the Pacific.

CCL (cm)	Australia		Colombia		Fiji		Galapagos^b^		Hawaii		Mexico^b^	
	cm year^−1^	*n*	cm year^−1^	*n*	cm year^−1^	*n*	cm year^−1^	*n*	cm year^−1^	*n*	cm year^−1^	*n*
≤ 50.0	0.8 ± 0.6	4	1.2 ± 0.3	6	1.7 ± 0.4	49	0.3 ± 0.3	3	1.1 ± 0.5	4	—	—
50.0–59.9	1	1	1.5^a^	6	1.9 ± 0.1	115	0.4 ± 0.5	14	1.1 ± 0.4	21	1.0 ± 0.7	2
60.0–69.9	±2.0	14	0.9 ± 0.2	13	1.3 ± 0.4	64	0.03 ± 0.4	13	1.4^a^	7	1.4 ± 1.3	4
70.0–79.9	1.5 ± 0.7	15	0.2	1	1.5 ± 0.7^c^	23	0.03 ± 0.3	10	n/a	1	1.2 ± 0.7	6
80.0–89.9	—		—		—		0.2	1	—		1.9 ± 1.1	7
> 90.0	—		—		—		—		—		1.0 ± 0.8	2

*Note:* Sites include Australia (Limpus and Chaloupka 1997), Colombia (Sampson et al. 2015), Fiji (this study), Galapagos (Zárate et al. 2015), Hawaiian Islands (Balazs and Chaloupka 2004), and Gulf of California, Mexico (Seminoff et al. 2002). Means are followed by ± standard error. *n* represents total individual size for each study.

^a^ No mean variance reported.

^b^ All values straight carapace length (SCL).

^c^ Mean growth for all green turtles w/CCL > 70.0.

### 4.4 Implications for Life‐Stage Duration

In addition to raising attention to the environmental conditions that Fijian green turtles face, knowledge about somatic growth rates, especially when evaluated across size classes, can provide insight about the stage‐ or residency duration of local green turtles. Using the mean growth rates for each 10‐cm size class of turtles, at each of the three foraging areas (excluding the four putative adults at Kanacea Island), and the overall size range per site, we estimated the total time required for turtles to reach the maximum observed body size for each habitat. Overall, stage duration for each 10‐cm size class group was estimated at 5.7–9.2 years for turtles in the ≤ 50.0 cm CCL size class, 4.9–5.4 years for the 50.1–59.9 cm CCL size class, 5.8–10.8 years for the 60.0–69.9 cm CCL size class, and 15.5–42.5 years for the ≥ 70.0 cm CCL size class. Based on these size interval‐specific values, the residency duration for turtles at each foraging location was estimated at 52.9 years for Kanacea Island (max 104.0 cm CCL), 22.0 years for Makogai Island (max 77.7 cm CCL), and 18.5 years for Yadua Island (max 77.8 cm CCL). Although more data are required to substantiate this estimate, it is apparent that green turtles may require decades in these regional foraging habitats before transitioning to new foraging areas as large immature and/or adult turtles.

## 5 Summary

In summary, the Fijian archipelago hosts dynamic and important foraging areas and developmental habitat for green turtles originating from several different nesting beaches in the South Pacific (Craig et al. 2004; Batibasaga et al. 2006; Piovano et al. 2019). Green turtle growth dynamics underscore the effect of local habitat structure, resource availability, and recruitment and dispersal patterns on green turtle growth within the archipelago and highlight that continued conservation efforts and research studies are essential for comprehensive and effective green turtle management. We believe this study provides important growth data that will guide and inform conservation management decisions in the future. Accordingly, we recommend research in three principal areas: (1) the maintenance and preservation of long‐term demographic datasets from this and other regional studies to strengthen future population assessments; (2) the expansion of systematic surveys of green turtles and their habitats across additional sites within the archipelago; and (3) further investigation into local green turtle movement ecology, with specific emphasis on recruitment dynamics, dispersal pathways, and ontogenetic shifts in physiology and resource utilization.

## Author Contributions

**Garrett E. Lemons:** data curation (lead), formal analysis (lead), methodology (lead), project administration (equal), visualization (lead), writing – original draft (lead). **Calandra N. Turner Tomaszewicz:** formal analysis (supporting), writing – review and editing (supporting). **Shritika Prakash:** data curation (supporting), investigation (supporting), methodology (supporting), writing – review and editing (supporting). **Katy Miller:** methodology (supporting), resources (supporting), writing – review and editing (supporting). **Jeffrey A. Seminoff:** methodology (supporting), supervision (supporting), writing – original draft (supporting), writing – review and editing (supporting). **Susanna Piovano:** conceptualization (lead), data curation (equal), funding acquisition (lead), investigation (lead), methodology (lead), resources (lead), writing – original draft (equal), writing – review and editing (supporting).

## Ethics Statement

All animal capture, handling, and sampling and were done following local permit guidelines and international IACUC standards.

## Conflicts of Interest

The authors declare no conflicts of interest.

## Data Availability

The authors declare that data used in this study were made available for the peer review process as a supplemental file in compliance with data sharing and peer review policies.
